# Effects of High-Pressure Homogenization Treatment on the Development of Antioxidant *Zanthoxylum bungeanum* Leaf Powder Films for Preservation of Fresh-Cut Apple

**DOI:** 10.3390/foods13010022

**Published:** 2023-12-20

**Authors:** Fuli Li, Fan Zhang, Ruixian Chen, Zexiang Ma, Hejun Wu, Zhiqing Zhang, Shutao Yin, Man Zhou

**Affiliations:** 1 College of Food Science, Sichuan Agricultural University, No. 46, Xin Kang Road, Ya’an 625014, Chinacrx2023218026@163.com (R.C.); zqzhang721@163.com (Z.Z.); 2 Institute of Modern Agricultural Industry, China Agricultural University, Chengdu 611430, Chinadogpossible@163.com (Z.M.); 3 College of Science, Sichuan Agricultural University, No. 46, Xin Kang Road, Ya’an 625014, China; hejunwu520@163.com

**Keywords:** high-pressure homogenization, *Zanthoxylum bungeanum* leaf, fresh-cut apple packaging, antioxidant

## Abstract

This study determined that *Zanthoxylum bungeanum* leaves (ZBLs) are rich in functional components such as cellulose, protein, flavone, and polyphenols. Therefore, they were used as the main raw material, with sodium alginate as a thickener and glycerol as a plasticizer, to investigate the preparation of active films from ZBL powder through high-pressure homogenization (HPH). The physical, optical, mechanical, and antioxidant properties of the films were evaluated, and their application in preserving fresh-cut apples was examined. The results showed that the optimal concentration of ZBL powder was 1.5% under a 30 MPa HPH treatment. The resulting HPH-treated films exhibited a denser microstructure and improved water vapor barrier properties and mechanical strength. Compared to the films without HPH treatment, the tensile strength increased from 4.61 MPa to 12.13 MPa, the elongation at break increased from 21.25% to 42.86%, the water vapor permeability decreased from 9.9 × 10^−9^ g/m·s·Pa to 8.0 × 10^−9^ g/m·s·Pa, and the transparency increased from 25.36% to 38.5%. Compared to the control group, the fresh-cut apples packaged with the HPH-treated ZBL active films exhibited effective preservation of apple quality during a five-day period at 4 °C and 70% humidity, showing better preservation effects than the other groups. In conclusion, the use of HPH treatment in developing novel biopolymer active films from ZBL powders with enhanced properties holds potential for various applications.

## 1. Introduction

Petroleum-based plastic packaging materials, due to their high chemical stability and extremely low biodegradability, combined with the inadequate treatment of plastics by human activities, low recycling rates, and many other factors, can cause severe environmental pollution. With increasing concerns about food safety, ecology, and environmental issues, research on biodegradable films made from natural biopolymers as alternatives to traditional petroleum-based materials has experienced rapid expansion [1]. During agricultural product processing, a large amount of processing by-products is often generated. These by-products are not fully utilized and are frequently wasted and discarded, leading to potential environmental issues and the waste of value-added products. It has been reported that these by-products, such as leaves, bran, and peels, contain abundant biopolymers (e.g., cellulose, proteins, and polysaccharides) and bioactive compounds (e.g., polyphenols and phytosterols) with high added value, making them potential raw materials for the preparation of biodegradable packaging.

The solid residues of plant food by-products (such as leaves, bran, fruit husks, and nut shells) can be made into biodegradable packaging films by grinding them into powder and then processing them to prepare basic film-forming materials. Suitable thickeners, plasticizers, and cross-linking agents are then added to reduce the viscosity and brittleness of the films and improve their performance to meet the requirements of packaging materials [2,3,4]. Additionally, due to the presence of functional components in plant-based processing by-products, the prepared packaging films may possess active characteristics. Previous research by Kam et al. [5] reported on incorporated durian leaf extract into gelatin-based films, showing that the gelatin-based films with a 0.5% mass fraction of durian leaf crude extract showed a three-fold increase in free radical scavenging capacity, demonstrating that the addition of durian leaf extract enhanced the antioxidant functionality of the packaging films.

China is the country with the most comprehensive cultivation area, yield, and variety of *Zanthoxylum bungeanum* (also known as Chinese prickly ash). The “large branch picking” method of its cultivation has made ZBLs one of the main processing by-products. ZBLs are rich in nutrients such as cellulose, proteins, vitamins, and fats. They also contain minerals such as calcium, iron, and phosphorus, making them highly valuable for consumption [6]. The abundant chemical composition of ZBLs, including volatile oils, flavones, polyphenols, polysaccharides, and amides, gives them antioxidant and antibacterial activities, making them valuable for medicinal purposes. Currently, domestic and international research on ZBLs mainly focuses on the extraction and isolation of functional components; however, the process is costly and complex. ZBLs are rich in functional components such as cellulose, proteins, flavonoids, and polyphenols. Exploring their application in the preparation of biologically active and biodegradable films opens up a new direction and holds important significance for the recycling and utilization of ZBLs in terms of economic benefits and environmental protection [7,8].

Due to the presence of insoluble dietary fibers such as cellulose and lignin in plant-based food by-products, biodegradable active packaging films prepared using them as the main raw materials may have larger particle sizes in the pulp without any special treatment. This can result in the apparent poor structure, mechanical properties, and barrier performance of the prepared biopolymer films, limiting their application development [9]. HPH, as a commonly used physical modification technique in fluid material processing, can effectively improve the uniform dispersion of the pulp by significantly influencing the rheological properties of the fluid pulp. The high pressure reduces the particle size and disrupts the cells in the film pulp, leading to an increase in the surface area of suspended particles, as well as the release of substances such as pectin and proteins. This enhances the interaction between the macromolecules in the film and alters its properties [10,11,12]. Wu et al. [13] used HPH at different pressure levels (0–80 MPa, fixed for 10 cycles) to prepare edible films based on pomelo peel. Their results showed that HPH reduced the elastic modulus and viscosity of the pomelo peel film-forming solution, which is ideal for obtaining a uniform biopolymer film. Therefore, it is necessary and meaningful to use simple treatments such as HPH to optimize the preparation of biopolymer films based on ZBL powder. To the best of our knowledge, there have been no reports on the use of leaf powder with a high content of cellulose as a treatment for film applications.

In summary, this study utilized ZBL powder as the raw material, sodium alginate as the thickener, and glycerol as the plasticizer to prepare active films based on ZBLs using a casting method. Since the suspension of ZBL powder had large particles and poor dispersion, HPH technology was employed to process the suspension. The effects of the ZBL powder content and HPH treatment on the film-forming properties of the leaf powder were investigated. The improvement in the overall performance and appearance quality of the active films based on ZBLs were evaluated through an examination of the morphology, optical properties, barrier properties (water solubility, water vapor permeability, water contact angle, and oxygen transmission rate), and mechanical properties of the biopolymer films. Finally, the application of the active films based on ZBL powder in the preservation of fresh-cut apples was explored. This research is believed to provide important technical references for the value-added and functional utilization of plant-based food byproducts rich in cellulose and natural active components, as represented by ZBLs.

## 2. Materials and Methods

### 2.1. Materials

No obvious pollution or pest disease-damaged *Zanthoxylum bungeanum* leaves were chosen and purchased from a market in Ya’an City, Sichuan Province. The leaves were washed with distilled water and then air-dried in a well-ventilated area at room temperature. The dried ZBLs were ground in an ultrafine grinder (YSC-701, Beijing Yanshan Zhengde Machinery Equipment Co., LTD, Beijing, China), and the resulting powder was sieved through a sieve with a pore diameter of 74 μm. The ZBL powder was then packed in sealed plastic bags and stored in a refrigerator at 4 °C and a humidity of 70% for further use. The main components and contents of the active substances in the ZBL powder are shown in Table 1. Refer to the Supplementary Materials for measurement methods.

### 2.2. Preparation of Zanthoxylum bungeanum Leaf-Based Films

An appropriate amount of ZBL powder was weighed and dissolved in 100 mL of distilled water. The mixture was homogenized in a fruit and vegetable homogenizer (JYL-G12E, Joyoung Co., Ltd., Dongguan city China) at a speed of 15,000 rpm for 5 min to obtain a uniform dispersion. The homogenized suspension was then subjected to high-pressure homogenization (GJJ-0.03/100, Shanghai Noni Light Industrial Machinery Co., LTD, Shanghai, China). For every 100 mL of the homogenized suspension, a certain amount of plasticizer (glycerol, 0.6%) and thickener (sodium alginate, 0.5%) was added, and the mixture was stirred at 50 °C and 1500 rpm for 30 min until fully dissolved, resulting in a uniform film-forming solution of ZBL powder. The film-forming solution was poured into a vacuum filtration bottle to remove air bubbles. Then, 200 mL of the suspension was taken from each film and cast on a plastic plate measuring 25 cm × 25 cm × 0.5 cm. The cast film was dried at 50 °C for 6–8 h and then peeled off. The obtained film was placed in a constant temperature and humidity chamber at 25 °C and 55% relative humidity for 48 h to reach equilibrium (HD-E702-100, Haida International Instruments LTD, Dongguan city, China). The various properties of the film were then measured. The concentration of glycerol and sodium alginate were determined to be 0.6% and 0.5%, respectively, while the homogenization pressure was set at 30 MPa for 10 cycles. The effects of different concentrations of ZBL powder (0.5%, 1.0%, 1.5%, 2.0%, and 2.5%) on the properties of the active films based on ZBLs were investigated.

### 2.3. Characterization of the Films

#### 2.3.1. Film Thickness

According to the national standard, “Plastic Film and Sheet Thickness Determination Mechanical Measurement Method” (GB/T6672-2001) [14], the film thickness was measured using a portable digital thickness gauge (EXPLOIT, Yiwu City Development Hardware Co., LTD, Yiwu city, China). Five random points were measured, and the average value was taken. The unit of measurement was millimeters (μm).

#### 2.3.2. Mechanical Property Measurement

The mechanical properties of the film, including tensile strength (TS, MPa) and elongation at break (EAB, %), were determined using an automatic tensile testing machine (HD-A821-1, Dongguan Haida Instrument Co., LTD, Dongguan city, China), in accordance with the method specified in “GB/T1040-2006 Determination of Mechanical Properties of Plastics” [15]. Prior to testing, the film was conditioned at 25 °C and 50% relative humidity for 48 h. During the testing, the film was cut into dimensions of 80 mm × 15 mm. The initial gauge length was set at 40 mm, and the testing speed was set at 60 mm/min. The measurements were repeated five times, and the average values were recorded.

#### 2.3.3. Color Measurement

Following the experimental method of Ghasemlou et al. [16], five random points were selected within each film sample. The color difference of the ZBL film was measured using an automatic spectrophotometer (CR-400, Konica Minolta, Shanghai, China). The color difference, Δ*E* (degree of color change), was calculated according to Formula (1).

(1) $$∆E=\sqrt{{\left(L^{*}-L\right)}^{2}+{\left(a^{*}-a\right)}^{2}+{\left(b^{*}-b\right)}^{2}}$$

In the formula, *L*, *a*, and *b* are the original color parameter values of the as-prepared film, and L*, a*, and b* are the color parameters of the film after discoloration (at different pH levels).

#### 2.3.4. Transparency Measurement

Referring to the test method for the transparency of transparent plastics in GB/T 2410-2008 [17], the sample film was cut into 5 cm × 5 cm specimens, and a WGW photometric mist instrument (WGW, Shanghai Yiden physical Optical Instrument Co., LTD, Shanghai, China) was used for measurement. Five samples were tested for each group, and the average value was taken as the result, with the unit being %.

#### 2.3.5. Water Vapor Permeability

According to the test methods for the gas permeability of plastic films and sheets in GB/T 1038-2000 [18] and the test method for the water vapor transmission rate of plastic films and sheets in GB/T 1037-1988 [19], a water vapor transmission rate testing instrument (W3/031, Jinan Languang electromechanical Technology Co., LTD, Jinan city, China) and its software were used to calculate the water vapor permeability coefficient (WVP, g·m/m^2^·s·Pa).

#### 2.3.6. Infrared Spectral Analysis

The film samples were analyzed using a Fourier transform infrared spectrometer (Thermo Fisher Scientific, Waltham, MA, USA) with an ATR accessory. The scanning range was from 4000 to 650 cm^−1^, with 64 scans at a resolution of 4 cm^−1^ [20].

#### 2.3.7. Scanning Electron Microscopy

The ZBL-based film was cut into 4 mm × 4 mm specimens, dried at 30 °C for 10 h, and cryo-fractured with liquid nitrogen. When using the scanning electron microscope (ZEISS Sigma 300, Jena city, Germany), the film was fixed on a metal mount and sputter-coated under a vacuum. The electron beam acceleration voltage for the sample scanning was 20 kV [21].

#### 2.3.8. Water Contact Angle

The contact angle of the ZBL-based film was measured by an optical contact angle tester (Theta Flex, Shanghai IFEisi Precision Instrument Co., LTD, Shanghai, China) at room temperature. During the test, the equilibrium contact angle of a droplet on a solid plane was photographed every 5 s. Each film was photographed at least five times to reduce errors [22].

#### 2.3.9. DPPH Free Radical Scavenging Ability Measurement

The method of Burt et al. [23] was used with slight modifications. A total of 10 mL of ethanol solution was mixed with 0.1 g of the film to dissolve and mix it thoroughly. Then, 1 mL of the extract was mixed with 2 mL of 0.1 mmol/L DPPH ethanol solution and kept in a dark room for 30 min. The absorbance was measured at 517 nm using an ultraviolet spectrophotometer (UV-3100PC, Shanghai Mepuda Instrument Co., LTD, Shanghai, China). Three experiments were conducted for the ZBL-based film, and the average value was calculated. The calculation is shown in Formula (2).

(2) $$DPPH(\%)=\frac{ADPPH-A_{S}}{ADPPH}\times 100$$

In the formula, *DPPH* refers to the *DPPH* radical scavenging rate, *ADPPH* refers to the absorbance value of the *DPPH* ethanol solution at 517 nm, and *A_S_* refers to the absorbance value of the ZBL-based film and *DPPH* ethanol mixture at 517 nm.

#### 2.3.10. ABTS Cation Radical Scavenging Capacity Determination

The antioxidant capacity of the film was evaluated by the scavenging of ABTS cation radicals method [24]. A 10 mL ethanol solution was mixed with 0.1 g of the film to dissolve and mix it thoroughly. The *ABTS^+^* solution was prepared by mixing 7 mmol/L ABTS stock solution with 2.45 mmol/L potassium persulfate solution at a ratio of 2:1 and letting it stand in the dark overnight at room temperature. Before measurement, the *ABTS^+^* solution was diluted with ethanol to obtain an absorbance value of 0.700 ± 0.002 at 734 nm (*A*_0_). Then, 0.2 mL of the extract and 8 mL of the *ABTS^+^* solution were mixed in the dark for 6 min, and the absorbance was measured at 734 nm (*A*_1_). The calculation is shown in Formula (3).

(3) $$ABTS^{+}(\%)=\frac{A_{0}-A_{1}}{A_{0}}\times 100$$

#### 2.3.11. Ferrous Ion Reducing Power Determination

The TPTZ working solution should be freshly prepared before use, and its preparation method [25] was as follows: a 300 mmol/L acetate buffer solution with a pH of 3.6, a 10 mmol/L TPTZ solution dissolved in a 40 mM HCl solution, and a 20 mmol/L ferric chloride solution were mixed in a volume ratio of 10:1:1. The mixture was preheated to 37 °C before use. A 10 mL ethanol solution was mixed with 0.1 g of the film to dissolve and mix it thoroughly. Then, 0.1 mL of the film extract solution was added to a test tube, and 2 mL of freshly prepared TPTZ working solution preheated to 37 °C was added. The mixture was mixed well and incubated in a 37 °C water bath for 30 min. Ethanol was used as the blank control. The absorbance was measured at 593 nm using an ultraviolet spectrophotometer (UV-3100PC, Shanghai Mepuda Instrument Co., LTD, Shanghai, China). Three parallel measurements were performed. A standard curve was established using FeSO_4_, with a regression equation of y = 0.0006x − 0.0334 (200–1000 μM, R^2^ = 0.9988). The ferrous ion-reducing power of the film extract samples was expressed as millimoles of FeSO_4_ equivalent per gram of sample.

### 2.4. Application of ZBL Film in Freshly Cut Apple Packaging

#### 2.4.1. Sample Preparation

Locally purchased, fresh, and free of visible insect infestation Daliang Mountain ugly apples were selected, cleaned, pitted, and uniformly sliced. The sliced apples were randomly divided into four groups and placed flat in uncovered polypropylene containers. The first group was left untreated, and the apple slices were exposed to air as a blank control. The second, third, and fourth groups were sealed with a homogeneous ZBL-based film, non-homogeneous ZBL-based film, and ZBL powder-free film, respectively. Each group consisted of six containers, with approximately six apple slices per container. The samples were stored in a refrigerator at a temperature of 4 °C and a humidity of 70%. The quality parameters of the samples were measured daily.

#### 2.4.2. Weight Loss Rate

The weight loss rate, weight loss rate determination [26], was measured using the weighing method (FA2004N, Shanghai Jinghai Instrument Co., LTD, Shanghai, China), and calculated according to Formula (4).

(4) $$Rate(\%)=\frac{M_{0}-M_{t}}{M_{0}}\times 100$$

In the formula, *M*_0_ is the initial mass of the sample, in grams; *M_t_* is the mass of the sample at time *t* of storage, in grams.

#### 2.4.3. Determination of Browning Index, Hardness, Total Soluble Solids, Titratable Acid Content, and pH

##### Browning Index (BI) Determination

The surface color of apple slices, the BI determination [27], was measured using an automatic color difference meter (CR-400, Konica Minolta, Shanghai, China). This was used to indicate the degree of browning of the apple. The browning index (BI) was calculated using Formula (5), where *L* represents brightness, a represents the red-green value, and b represents the yellow-blue value.

(5) $$BI=100\times \left(\frac{a+1.75L}{5.645L+a-3.012b}-0.31\right)÷0.172$$

In the formula, *L*, *a*, and *b* are the original color parameter values of the as-prepared film.

##### Hardness Determination

A CY-4 digital fruit hardness tester, with units in kg/cm^2^, was used to determine hardness. The test was conducted following the instructions of the instrument. Ten random apple slice samples were selected under each storage condition, and the hardness of the fresh-cut apples was measured to obtain an average value [28].

##### Total Soluble Solid Content Determination 

Random apple slice samples under each storage condition were weighed and ground with a certain amount of distilled water. After filtration, the filtrate was collected [29]. The determination of the total soluble solids was conducted using a handheld refractometer, with units in Brix.

##### Titratable Acid Content Determination

The titratable acid content of the fresh-cut apple slices was determined using the acid–base titration method according to the national standard [30].

##### pH Determination

The pH meter of the PHS-25 model was used for pH determination. Before measuring, the instrument was calibrated to ensure the accuracy of the measured value.

### 2.5. Statistical Analysis

The experimental data were processed using SPSS 26.0 software. The data are expressed as the mean ± standard deviation. The Duncan multiple comparison test (*p* < 0.05) was used for significance analysis, and OriginPro 2021 software was used for graphing.

## 3. Results and Analysis

### 3.1. Screening of the Amount of ZBL Powder Added

#### 3.1.1. Influence of the Amount of ZBL Powder Added on the Thickness and Optical Properties of ZBL Film

Film thickness is an important characteristic that affects its basic properties, such as optical properties, barrier properties, and mechanical properties. As shown in Table 2, the thickness of ZBL film significantly increases with the increase in the amount of ZBL powder added. This may be due to the fact that, as the proportion of ZBL powder added increases, the film-forming solution contains more ZBL powder particles, leading to an increase in film thickness [31].

Transparency and brightness directly affect the overall appearance of packaged products and consumer acceptance. Moreover, a lower transparency reduces the penetration of light through the packaging film onto the inner food, effectively blocking the entry of heat [32]. From Table 2, it can be observed that with an increase in the amount of ZBL powder added, the ΔE gradually increases, and the transparency of the film decreases significantly. When the amount of ZBL powder added increases from 0.5% to 2.5%, the transparency of the film decreases from 65.22% to 19.90%. When the concentration of ZBL powder is 2.5%, the color reaches 1175 due to the characteristics of the ZBL powder, compared with 1%, the color increased by 97% and the transmittance is the lowest. The poor optical properties of ZBL film can negatively affect consumer preferences. Additionally, as indicated by the L-value of the color difference, the brightness of the film gradually darkens with an increase in the amount of ZBL powder added. This is because as the amount of ZBL powder added increases, aggregation occurs between large molecular substances, like cellulose, in the film-forming solution, thereby disrupting the network structure of the film and further hindering light transmission. Moreover, as the film thickness increases, light transmission becomes more difficult. Therefore, when considering the increase in film thickness, decrease in transparency, and decrease in brightness with the increasing amount of ZBL powder added, other indicators need to be considered for the optimal concentration of ZBL powder to be added.

#### 3.1.2. Influence of the Amount of ZBL Powder Added on the Moisture Content (MC) and Water Solubility (WS)

**Figure 1:**
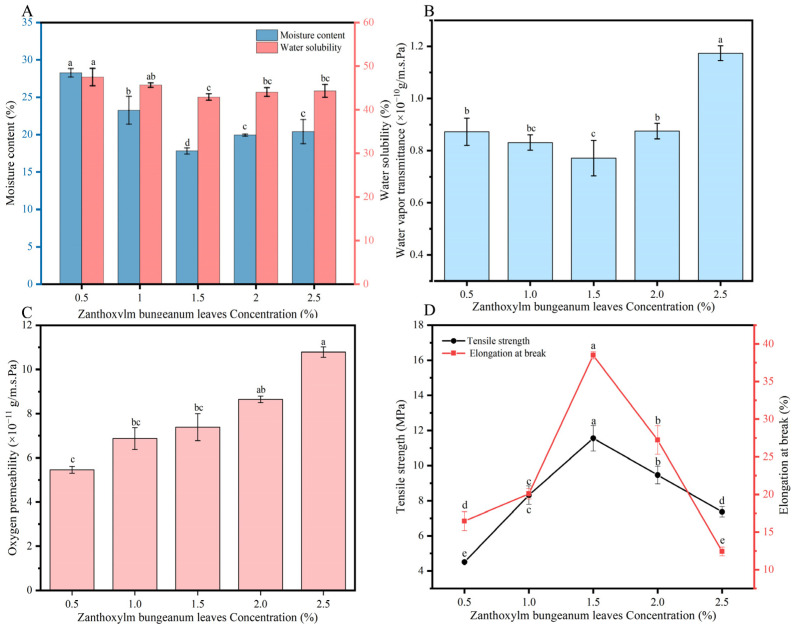
Effect of the addition of *Zanthoxylum bungeanum* leaf powder on the film moisture content and water solubility (**A**), water vapor transmission coefficient (**B**), oxygen transmission rate (**C**), and mechanical properties (**D**). Different letters have significant differences (*p* < 0.05).

As shown in Figure 1A, ZBL film has relatively high moisture content (MC) and water solubility (WS). With the increase in ZBL powder added, both the MC and WS of ZBL film show a decreasing and then increasing trend. When the addition of ZBL powder is 1.5%, the MC and WS of ZBL film are the lowest. This change is due to the HPH treatment that breaks down the macromolecular structure of the fibers. At low concentrations, the disrupted cellulose may expose hydrophilic groups, enhancing moisture absorption. At high concentrations, the increase in pulp concentration may lead to more insoluble dietary fibers (such as cellulose and lignin), causing particle aggregation and reducing the moisture content and water solubility of the film [33]. Typically, potential commercial packaging films require hydrophobic or water-resistant properties to protect food from moisture impact or loss [34], but for certain specific applications, a high WS may be desirable. For example, when an edible film is intended to melt/dissolve during cooking or when coming into contact with hot food components, water solubility can prevent sensory changes [35].

#### 3.1.3. Influence of the Amount of ZBL Powder Added on the Water Vapor Permeability (WVP)

Water vapor permeability (WVP) is an important indicator for evaluating the performance of packaging films in order to prevent or reduce the moisture transfer between packaged products and the surrounding environment. Its magnitude depends on many factors, such as film thickness, relative humidity of the environment, and the relative proportions of the components in the solution formulation. Therefore, a low WVP is an ideal property for packaging films. As shown in Figure 1B, when low concentrations (0.5–2.0%) of ZBL powder are added, the WVP values are generally low. With an addition of 1.5% ZBL powder, the WVP value is the lowest at 0.77 × 10^−10^ g·m/m^2^·s·Pa, showing the best water barrier performance. This may be because films with lower concentrations of ZBL powder are thinner and have a more compact structure and smaller pores after HPH treatment, resulting in lower WVP values [36]. When the concentration of ZBL powder is 2.5%, the film becomes thicker, and even higher concentrations result in more cellulose, causing a looser structure, increased pores, and cracks during film formation, which significantly increases the WVP value.

#### 3.1.4. Influence of the Amount of ZBL Powder Addition on the Oxygen Permeability

The oxygen permeability coefficient is one of the important indicators for evaluating film performance. To extend the shelf life of food and prevent spoilage and deterioration caused by factors such as air, films with high barrier properties, i.e., low oxygen permeability, need to be selected. As shown in Figure 1C, with the increase in ZBL powder added, the oxygen permeability coefficient of ZBL film increases. The oxygen permeability coefficient of the film mainly depends on its structure, and the denser the structure, the better the oxygen barrier performance. With increasing ZBL powder concentration, the oxygen permeability coefficient of the film increases to 10.78 × 10^−11^ g/m·s·Pa. This is because an excessive amount of ZBL powder particles can cause aggregation and microvoids on the film surface, affecting the performance of the ZBL-based film.

#### 3.1.5. Influence of the Amount of *Zanthoxylum bungeanum* Leaf Powder Added on the Mechanical Properties of ZBL Film

Mechanical properties represented by tensile strength (TS) and elongation at break (EAB) are important for maintaining packaging integrity when withstanding external stress. As shown in Figure 1D, with the increase in ZBL powder added, both the TS and EAB initially increased and then decreased. When the pulp concentration reaches 1.5%, the film exhibits good mechanical properties, with the TS and EAB reaching 11.56 MPa and 38.49%, respectively. Regarding mechanical properties, as the pulp concentration gradually increases, the interaction between the large molecules of film components strengthens due to molecular bonding, forming a good rigid network structure during film formation, resulting in a denser internal structure of the film, thus, increasing the TS and EAB. However, when the pulp concentration is too high, the increased content of film-forming components leads to an aggregation of excess cellulose in the pulp, which disrupts the film network structure, resulting in decreased strength and ductility and easy rupture [37]. Therefore, considering all the performance indicators mentioned above, 1.5% is selected as the optimal concentration of added ZBL powder.

### 3.2. Influence of HPH on the Characteristics of Zanthoxylum bungeanum Leaf-Based Films

#### 3.2.1. Influence of HPH Treatment on Morphology, Thickness, and Optical Characteristics

**Figure 2:**
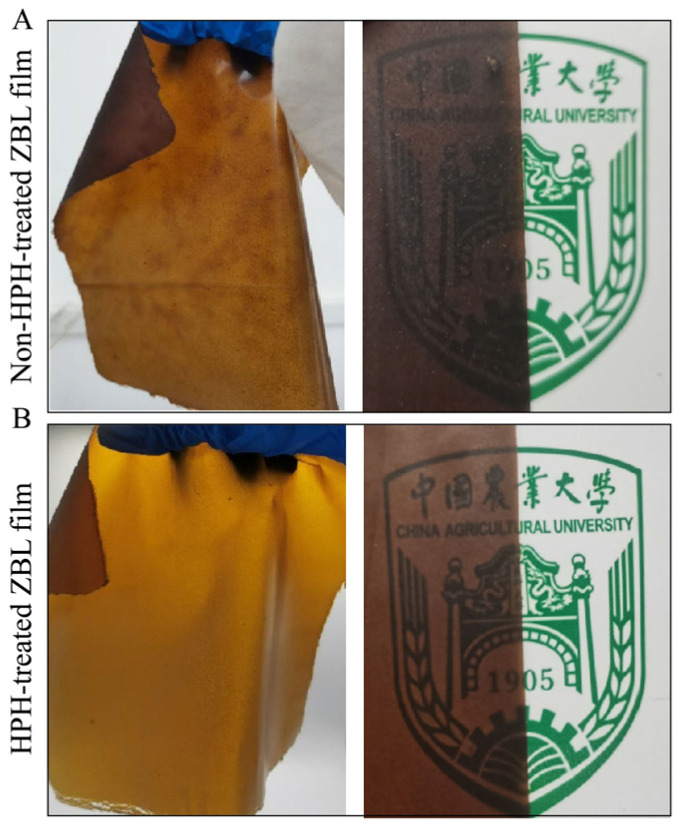
The appearance of *Zanthoxylum bungeanum* leaf film treated without high-pressure homogenization (**A**) and that with high-pressure homogenization (**B**).

**Figure 3:**
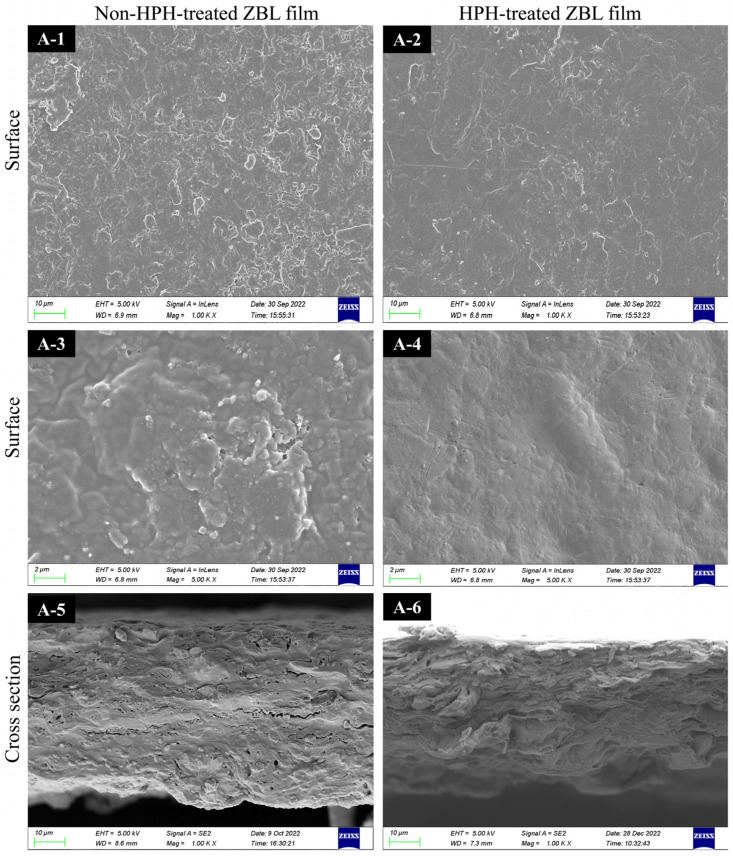
SEM images of *Zanthoxylum bungeanum* leaf film treated with and without high-pressure homogenization (HPH). The film surface without HPH (**A-1** and **A-3**), the film cross section without HPH (**A-5**), the film surface with HPH (**A-2** and **A-4**) and the film cross section with HPH (**A-6**).

From the images in Figure 2, it can be seen that the overall color of the film is yellowish brown. The mechanical breakdown and high-temperature effects of HPH cause the chlorophyll in ZBL powder to be oxidized and degraded, resulting in a yellow-brown color change of the ZBL film. In addition, it can be seen from the SEM in Figure 3, that the surface of ZBL-based film becomes smooth and has reduced roughness after HPH treatment. In cross-section, the pores are reduced and become more textured. In addition, the light transmittance increases by 13.14%, and the thickness decreases. 

#### 3.2.2. Enhancement of Mechanical and Water Barrier Properties of ZBL Film with HPH Treatment

As shown in Table 3, the film after HPH treatment exhibits improved mechanical and barrier properties. The tensile strength (TS) increases by 7.52 MPa, elongation at break (EAB) increases by 21.61%, and water vapor permeability (WVP) decreases by 0.19 × 10^−10^ g/m·s·Pa. The main reason for these improvements is that the HPH treatment reduces the particle size of ZBL powder, enhances the interaction between particles, and leads to a more uniform mixing of film components, resulting in a denser structure and better mechanical and barrier properties.

The moisture content of the film decreases by 6.28% after HPH treatment, while the water solubility increases by 6.05%. This is because the HPH process reduces the particle size of the ZBL powder and increases the soluble components, thereby reducing the moisture content and increasing water solubility.

The water contact angle (WCA) is commonly used to evaluate the hydrophobicity and surface-wetting properties of polymer films. As shown in Table 3, the water contact angle of the untreated film is 86.60°, which decreases to 78.32° after HPH treatment. Both values are below 90°, indicating that both films contain a small number of polar groups and exhibit slight hydrophilicity on the surface. A smaller water contact angle indicates a more hydrophilic film. From the SEM images in Figure 3, it can be observed that the surface of the ZBL-based film becomes smoother and has reduced roughness after HPH treatment, leading to a decrease in the water contact angle. The rough and uneven surface of the untreated film results in a larger water contact angle. Similar results have been observed in the preparation of methylcellulose/stearic acid blend films using ultrasound treatment [38].

#### 3.2.3. Infrared Spectral Analysis of HPH-Treated ZBL Active Film

**Figure 4:**
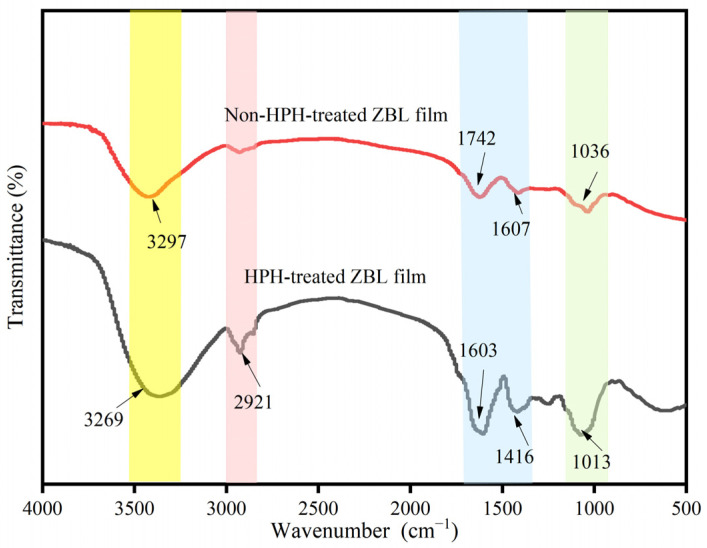
Infrared spectra of *Zanthoxylum bungeanum* leaf film treated with high-pressure homogenization.

As shown in Figure 4, HPH treatment did not change the infrared spectral structure of the film. No new peaks were generated, indicating that the functional group structure of the ZBL film did not change, and no derivative chemical reactions occurred. This suggests that HPH technology is purely a physical treatment [39]. The infrared spectrum of the ZBL film corresponds to the characteristic peaks of the main structures of the biopolymers, such as cellulose and proteins, in ZBLs [40,41].

The stretching vibration absorption peak of O-H is observed in the wavenumber range of 3100–3600 cm^−1^, which is a characteristic spectral band for all cellulose and carbohydrates. Due to the strong mechanical force from HPH, the stretching vibration of the -OH groups in the HPH-treated ZBL film redshifts from 3297.82 cm^−1^ to 3269.37 cm^−1^, indicating a stronger -OH bond formation [42,43]. The absorption peak at 2921.27 cm^−1^ corresponds to the stretching vibration of -CH_2_- in cellulose, and this peak is significantly enhanced after HPH treatment. The peak at 1603.59 cm^−1^ corresponds to the stretching vibration of C=C bonds. By comparison with the untreated film, it is found that the intensity of the absorption peak at 3269.37 cm^−1^ is significantly higher than that at 3297.82 cm^−1^, indicating enhanced hydrogen bonding interactions between the macromolecular components of the film matrix, resulting in a decrease in the number of -OH groups [44]. Due to the mechanical action and the temperature increase during the HPH process, the appearance of a new peak at 1270 cm^−1^ also indicates that the HPH induces physicochemical changes in certain substances.

The FTIR spectral analysis seems to reveal the impact of HPH treatment on the interaction of biopolymers in ZBL film, thereby ensuring the reliability of the microstructural characteristics of the edible film.

#### 3.2.4. Thermogravimetric Analysis of ZBL Active Film Treated by High Pressure Homogenization

**Figure 5:**
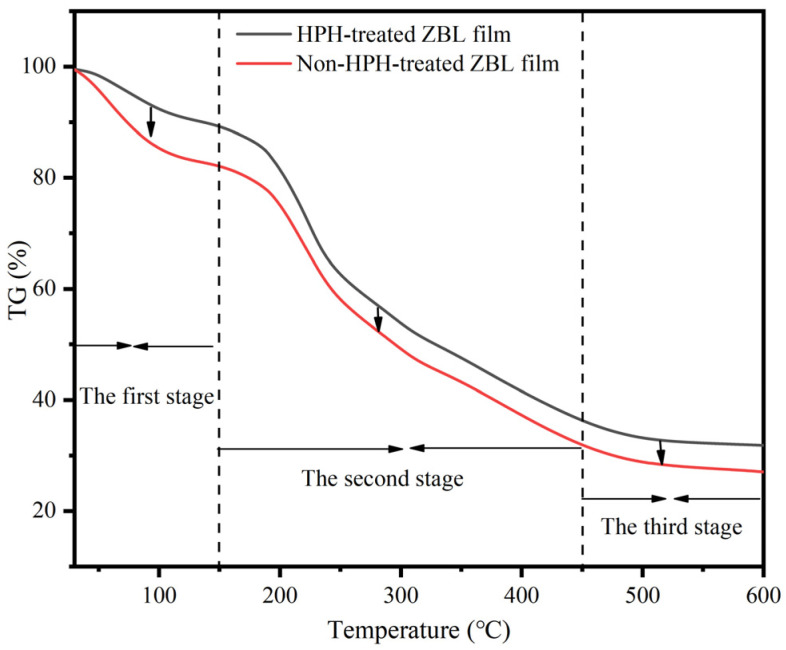
TGA diagram of *Zanthoxylum bungeanum* leaf film treated with high-pressure homogenization.

Thermogravimetric analysis (TGA) was conducted to further understand the effect of HPH on the interaction between the polymer structures in ZBL powder. The results showed that both films, with or without HPH treatment, exhibited three stages of mass loss. As shown in Figure 5, the first stage (30–120 °C) was attributed to the evaporation of water absorbed by the hydrophilic groups in the polymer structures of the films. It can be observed that the mass loss due to water adsorption was minimal, but the weight loss of the homogenized films was smaller than that of the non-homogenized films, indicating that the homogenized films had a lower water content. This corresponded to the trend of water content change shown in Figure 1A. The reason is that there were fewer interactions between the fewer free hydroxyl groups and water molecules after homogenization, as confirmed by infrared analysis, which demonstrated the presence of hydrogen bonding between the macromolecular components in ZBL powder. The second stage (150–450 °C) was attributed to the thermal degradation of biopolymers in the ZBL powder, mainly composed of cellulose, lignin, hemicellulose, and proteins. The third stage (450–600 °C) exhibited a decrease in film mass due to the thermal decomposition of carbon. This stage showed a relatively gentle slope, indicating a slower rate of mass loss.

Overall, HPH had little effect on the morphology of the TGA curves. However, the TGA curve of the homogenized film shifted towards higher temperatures, indicating an enhancement in the thermal stability of the film after HPH treatment. The SEM images in Figure 3 showed that the microstructure of the homogenized film was more compact with stronger molecular interactions, making it more difficult for the macromolecular chains to decompose. This was the reason behind the improved thermal stability. The experimental results suggested that the film formation temperature of the ZBL film should not exceed 150 °C to avoid damage to the film structure, which has practical implications for industrial applications [45].

#### 3.2.5. Antioxidant Analysis of HPH-ZBL Film

Free radicals induced by external factors are important contributors to the oxidative deterioration of food. The antioxidant capacity is an important indicator for evaluating active food packaging film. ZBL powder is composed of various components, and studies have shown that flavonoids, polyphenols, and tannins are related to the antioxidant properties of ZBLs [46]. In this experiment, the antioxidant capacity of ZBL powder and its film was evaluated from three aspects: DPPH radical scavenging activity, ABTS radical cation scavenging activity, and ferric ion-reducing power.

**Figure 6:**
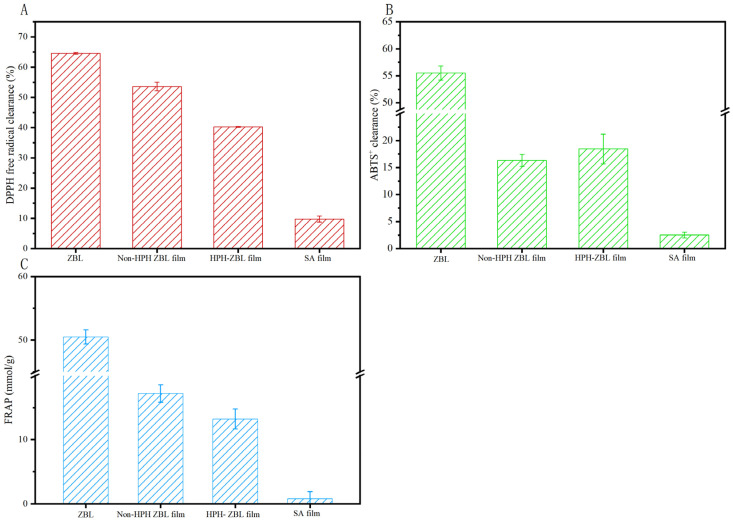
DPPH (**A**), ABTS clearance (**B**), and FRAP values (**C**) of *Zanthoxylum bungeanum* leaf film treated with high-pressure homogenization.

From Figure 6A,B, it can be seen that ZBL powder exhibited strong DPPH and ABTS radical scavenging activities, with a DPPH scavenging rate reaching 64.58% and ABTS radical cation scavenging activity reaching 55.54%. Compared with the antioxidant capacity of pure ZBL powder, the antioxidant capacity of the film was reduced, which may be because the preparation temperature had a slightly negative effect on the oxidation resistance. However, compared to the film without ZBL powder, the antioxidant capacity of ZBL film was improved. At the same time, the DPPH and FRAP of the HPH-ZBL film were both reduced compared with the film without high-pressure homogenization, because high-pressure homogenization may destroy some antioxidant substances in the leaves. From Figure 6C, the ferric ion-reducing power of ZBL powder was 50.50 mmol FeSO_4_/g. The ferric ion-reducing powers of the homogenized ZBL film and the non-homogenized ZBL film were slightly decreased, measuring 17.20 mmol FeSO_4_/g and 13.21 mmol FeSO_4_/g, respectively, while the film without ZBL powder was only 0.77 mmol FeSO_4_/g.

ZBL powder is composed of various components, and its antioxidant activity may be the result of the combined action of multiple active substances. Liu et al. [47] suggested that the high clearance rates of DPPH and ABTS radical cations were related to the effective release of polyphenols in the film.

### 3.3. The Effects of Zanthoxylum bungeanum Leaf-Based Films on the Preservation of Freshly Cut Apples

#### 3.3.1. Weight Loss Rate

**Figure 7:**
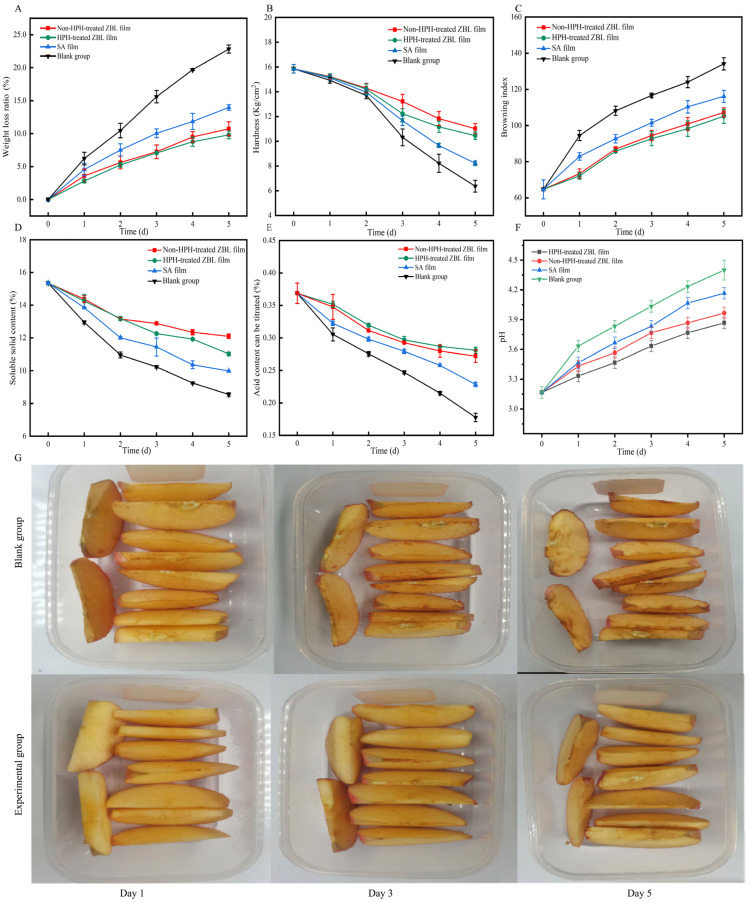
Weight loss rate of freshly cut apples during storage (**A**); hardness (**B**); browning (**C**); soluble solids (**D**); titrable acid content (**E**); pH change diagram (**F**); and fresh-cut apple fresh-keeping effect appearance chart (**G**).

Fresh-cut fruits and vegetables tend to undergo water loss during storage time, leading to an increase in the weight loss rate, which affects their commercial value. The ZBL film application in preserving fresh-cut apples was examined. As shown in Figure 7A, under the storage condition of 4 °C, the weight loss rates of fresh-cut apples packaged in different groups all showed an upward trend with the extension of storage time. This is mainly due to the loss of water and nutrients during the storage process caused by respiration, metabolism, and microbial corrosion [48]. The samples packaged with ZBL film exhibited the best weight loss rate, with a weight loss of 10.73% and 12.66% on the fifth day of storage. The weight loss rate of the sample with the thin film without the addition of ZBL powder increased and reached 17.74% on the fifth day. The weight loss rate of the control group increased significantly, reaching 23.88% on the fifth day of storage. The sodium alginate film without the addition of ZBL powder also inhibited the transpiration of fresh-cut apples to a certain extent, but the weight loss rate of the samples packaged with ZBL film was significantly lower than other treatment methods. This may be because the ZBL film effectively regulated the exchange of gases and moisture, further reducing the consumption of water in fresh-cut apples.

#### 3.3.2. Hardness

The loss of hardness is an important indicator for evaluating the quality and shelf life of fresh-cut fruits and vegetables. With the extension of storage time, the softening of the internal tissues of fruits and vegetables leads to aging and decay. As shown in Figure 7B, under the storage condition of 4 °C, the hardness of the fresh-cut apples in the experimental group was significantly higher than that of the control group samples. This is mainly because the control group lacked the protection of packaging film, resulting in more significant tissue aging and cell wall rupture caused by water loss and bacterial contamination in fresh-cut apples [49]. On the third day, the hardness curve showed a significant inflection point, and the hardness of all fresh-cut apples in all conditions started to decrease significantly due to fruit aging. This is mainly due to the consumption of a large amount of nutrients by microorganisms during the storage process, which increases moisture loss and leads to a softening of the surface and a decrease in the firmness of freshly cut apples [50]. On the fifth day, both the experimental group and the control group samples experienced a significant loss of hardness, but the hardness of the fresh-cut apple samples in the experimental group remained significantly higher than that of the control group.

#### 3.3.3. Browning

The degree of browning of fresh-cut apples during storage is represented by the browning index, with a higher value indicating a more severe browning degree [51]. The appearance changes during storage are shown in Figure 7G. As the storage time increased, the blank group experienced a more severe loss in quality and degree of browning compared to the experimental groups. As shown in Figure 7C, during the entire storage period, the browning indexes of the fresh-cut apples in all groups showed an upward trend, but the increase rate in the experimental group was significantly lower than that in the control group. On the fifth day, the browning index of the control group had already exceeded 130, which is 20.15% higher than that of the homogenized ZBL film group. The browning index of the samples packaged with the film without the addition of ZBL powder was higher than that of the samples packaged with the film containing ZBL powder. This may be related to the active ingredients contained in the ZBL film which have inhibitory effects on the formation of reactive oxygen species and related oxidative enzyme activity in apple fruits. The homogenization treatment had no significant effect on the browning index.

#### 3.3.4. Soluble Solids Content

The soluble solids content directly affects the taste of fresh-cut apples and can also directly reflect the changes in the quality and nutritional value of apples. It is an important indicator for evaluating fruit quality [52]. As shown in Figure 7D, under the storage condition of 4 °C, with the increase in storage time, the respiration and nutrient consumption of the fresh-cut apple tissues gradually increased, leading to a decrease in the soluble solids content [53], with the control group showing a significant downward trend. The soluble solids content in the fresh-cut apples packaged with ZBL active film remained relatively stable, staying within the range of 12.34 ± 0.16%. This may be because the active ingredients in the ZBL film partially inhibited the physiological metabolism of the apples, reducing respiratory consumption and decreasing sugar consumption.

#### 3.3.5. Titratable Acidity

The change in titratable acidity is greatly influenced by the metabolic rate of fruit. Organic acids are consumed during respiration, so acidity decreases during storage, which is also a major cause of fruit aging [54]. As shown in Figure 7E, with the extension of storage time, all four treatments showed a decreasing trend in titratable acidity. At the end of the storage period, the titratable acidity of fresh-cut apples in the control group (51.82%) was seen to have decreased significantly faster than that of the experimental group samples. The titratable acidity of the samples packaged without ZBL film decreased more rapidly, reducing by 43.38%. On the other hand, both the homogenized and non-homogenized ZBL active film reduced the titratable acidity by 26.22% and 23.80% respectively. This is because ZBL packaging can modify the internal atmosphere around fresh-cut apples, inhibit respiration, delay the utilization of organic acids, maintain a higher acid content in the fruit, and reduce nutrient consumption, thus prolonging shelf life.

#### 3.3.6. pH Value

As shown in Figure 7F, with the increase in storage time, the pH value of apples gradually increased, and the sour taste gradually decreased. After homogenization, the pH value of the pulp containing ZBL film increased slowly and was lower than that of the other groups. During the storage of apples, the cells digest oxygen and energy for respiration, and the internal starch is gradually converted into sugars, producing fructose and glucose, which reduces acidity and increases sweetness [55]. After homogenization, the oxygen transmission rate of ZBL film is low, which inhibits its respiration and slows down the metabolic process, which is consistent with the changing trend of titrable acid content mentioned above.

## 4. Conclusions

This study investigated the use of ZBL powder as the main raw material, sodium alginate as a thickener, and glycerol as a plasticizer to prepare active films. The effects of these films on the shelf life of fresh-cut apples were also examined. The optimal ZBL film with the best overall performance was obtained when the ZBL powder concentration was 1.5%, the sodium alginate amount was 0.5%, the glycerol concentration was 0.6%, and the homogenization pressure was 30 MPa. With an increase in ZBL powder concentration, the film surface became smoother, and the cross-section structure became denser with fewer gaps or cracks, as observed through SEM imaging. HPH helped generate ZBL particles with weaker interparticle interactions, resulting in biopolymer films with better uniformity and structural continuity. Therefore, these films exhibited lower water vapor permeability (WVP) and a higher tensile strength (TS) and elongation at break (EAB). Zanthoxylum leaves, rich in functional components such as cellulose, proteins, flavone, and polyphenols, have the potential to be used in the preparation of biodegradable active packaging films.

Homogenized ZBL films effectively delayed the deterioration of apple quality during a five-day storage period. In particular, after HPH, the film still had 89.72% weight on the fifth day, the lowest hardness loss was 30.52%, the browning index was lower than the blank group at 20.15%, the soluble solid content was still about 11%, and the titrable acid content was 35.7% higher than that of the blank group, demonstrating better preservation effects than other groups. ZBL-based active films prepared using the representative byproducts of fruit and vegetable processing, such as ZBLs, exhibited good mechanical and barrier properties and HPH treatment was shown to have the potential to develop novel biopolymer active films with enhanced performance.

## Figures and Tables

**Table 1:** The main components in *Zanthoxylum bungeanum* leaf.

*Zanthoxylum bungeanum* Leaf	Content
Moisture content	2.09 ± 0.13%
Lipid	1.47 ± 0.05%
Protein	19.98 ± 0.25%
Crude polysaccharide	7.33 ± 0.77%
Ash content	46.03 ± 1.11%
Total dietary fiber	20.48 ± 0.06 mg/g
Total phenol	26.82 ± 0.26 mg/g
Flavone	12.28 ± 0.22%

*Notes*: The contents of all components were expressed based on the dry weight of Zanthoxylum leaf powder stored at room temperature.

**Table 2:** Effects of different concentrations of *Zanthoxylum bungeanum* leaf on the properties of films.

ZBL Concentration (%)	Thickness (μm)	*L*	*a*	*b*	Δ*E*	T (%)
0.5	75 ± 0.01 ^e^	48.14 ± 0.12 ^a^	13.47 ± 0.29 ^c^	66.66 ± 0.34 ^a^	-	65.22 ± 1.08 ^a^
1.0	84 ± 0.00 ^d^	40.61 ± 3.61 ^b^	16.96 ± 1.78 ^b^	65.10 ± 1.93 ^a^	35.71 ± 0.38 ^d^	53.18 ± 0.89 ^b^
1.5	89 ± 0.00 ^c^	31.16 ± 1.31 ^c^	19.89 ± 0.66 ^a^	52.83 ± 2.11 ^b^	260.4 ± 0.79 ^c^	39.48 ± 1.74 ^c^
2.0	95 ± 0.00 ^b^	28.28 ± 1.01 ^d^	18.79 ± 0.22 ^a^	48.09 ± 1.66 ^c^	383.8 ± 0.21 ^b^	29.94 ± 0.44 ^d^
2.5	112 ± 0.00 ^a^	17.10 ± 0.88 ^e^	13.78 ± 0.12 ^c^	29.42 ± 1.09 ^d^	1175 ± 0.73 ^a^	19.90 ± 0.56 ^e^

*Notes:* Different letters in the same column indicate significant differences (*p* < 0.05). The meanings of each letter are as follows: *L*: lightness; *a*: hue; *b*: chroma; Δ*E*: chromatic aberration; and T: light transmittance.

**Table 3:** Effect of HPH treatment on the properties of ZBL films.

Film Type	Non-HPH-Treated ZBL Film	HPH-Treated ZBL Film
TS (MPa)	4.61 ± 0.13	12.13 ± 0.14
EAB (%)	21.25 ± 0.88	42.86 ± 0.66
WVP (×10^−10^ g/m·s·Pa)	0.99 ± 0.02	0.80 ± 0.01
T (%)	25.36 ± 0.48	38.50 ± 0.98
MC (%)	29.60 ± 0.65	23.32 ± 0.62
WS (%)	35.22 ± 0.15	41.27 ± 1.27
*L*	30.67 ± 1.09	31.10 ± 1.16
*a*	23.12 ± 0.12	24.19 ± 0.4
*b*	52.16 ± 1.76	52.92 ± 1.91
ΔE	-	0.96 ± 0.79
Water contact Angle	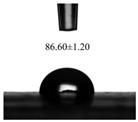	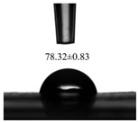

*Notes*: The meanings of each letter are as follows: HPH: high-pressure homogenization; ZBL: *Zanthoxylum bungeanum* leaf; TS: tensile strength; EAB: elongation at break; WVP: water vapor transmittance; MC: water content; and WS: water solubility.

## Data Availability

The data presented in this study are available from the corresponding author upon request.

## Supplementary Materials

The following supporting information can be downloaded at: https://www.mdpi.com/article/10.3390/foods13010022/s1. The testing method for the main components of *Zanthoxylum bungeanum* leaves.
