# Supplementary material for: Effects of High-Pressure Homogenization Treatment on the Development of Antioxidant Zanthoxylum bungeanum Leaf Powder Films for Preservation of Fresh-Cut Apple
Source: Foods. 2023 Dec 20;13(1):22. doi: 10.3390/foods13010022 (PMC10778247; doi:10.3390/foods13010022)
Supplement: Supplementary file 1 [file foods-13-00022-s001.zip › foods-2747929-supplementary.pdf]

foods

Effects of High-Pressure Homogenization Treatment on the Development of Antioxidant *Zanthoxylum bungeanum* Leaf Powder Films for Preservation of Fresh-Cut Apple

Fuli Li [1], Fan Zhang [2], Ruixian Chen [1], Zexiang Ma [2], Hejun Wu [3], Zhiqing Zhang [1], Shutao Yin [2,*] and Man Zhou [1,*]

1 College of Food Science, Sichuan Agricultural University, No. 46, Xin Kang Road, Ya'an 625014, China; crx2023218026@163.com (R.C.); zqzhang721@163.com (Z.Z.)
2 Institute of Modern Agricultural Industry, China Agricultural University, Chengdu 611430, China; dogpossible@163.com (Z.M.)
3 College of Science, Sichuan Agricultural University, No. 46, Xin Kang Road, Ya'an 625014, China; hejunwu520@163.com
* Correspondence: yinshutao@cau.edu.cn (S.Y.); zhouman@sicau.edu.cn (M.Z.)

The testing method for the main components of *Zanthoxylum bungeanum* leaves

1. *Determination of moisture content*

Refer to the determination method of direct drying specified in GB 5009.3-2016 Determination of Moisture in Food National Standard for Food Safety.

2. *Determination of ash content*

The total ash content in food was determined according to the determination method specified in GB 5009.4-2016 Determination of Ash Content in Food National Standard for Food Safety. It should be noted that the sample was weighed to be 2 g, accurate to 0.0001, and the rest of the steps can be found in the national standard method.

3. *Determination of protein content*

Refer to the automatic Kjeldahl nitrogen determination method specified in GB 5009.5-2016 Determination of Protein Content in Food National Standard for Food Safety. The sample was weighed to be 1 g, accurate to 0.0001, and the rest of the steps can be found in the national standard method.

4. *Determination of fat content*

The determination of fat in food was modified according to GB 5009.6-2016 Determination of Fat Content in Food National Standard for Food Safety. The acid hydrolysis method is used, in which the sample was weighed to be 2 g, accurate to 0.0001, and the rest of the steps can refer to the national standard method.

5. *Determination of dietary fiber*

Refer to GB 5009.88-2014 Determination of Dietary Fiber in Food According to National Food Safety Standards for the Determination of Dietary Fiber in Food.

6. *Determination of total phenol content*

The content of total phenol in *Zanthoxylum zanthoxylum* leaves was determined using the Folin–Ciocalteu method. A total of 0.1g of *Zanthoxylum bungeanum* leaves powder was weighed in a 25 mL centrifuge tube, then 10 mL 95% ethanol was added and shaken overnight, and then centrifuged for 3 min to obtain the supernatant to be measured. Next, 0.25 mL sample extract was absorbed into a 25 mL test tube using a pipette gun, then 1 mL Folin phenol reagent was added and shaken to stand for 30 s, 2 mL of 12% sodium carbonate solution was added, the volume was shaken to 25 mL, and then the absorbance was determined at a wavelength of 760 nm. Gallic acid aqueous solutions with concentrations ranging from 0 to 7 μg/mL were used as standard curves to determine the absorbance values of aqueous solutions with different concentrations at 760 nm, and the concentration of gallic acid was 0 as a blank. With the standard solution concentration (μg/mL) as the horizontal coordinate and absorbance as the vertical coordinate, the standard curve was drawn to obtain the equation $y=0.143x+0.0121$, $R^2=0.9972$, and then the total phenol content of *Zanthoxylum zanthoxylum* leaf was calculated via the standard curve.

7. *Determination of flavonoe content*

The content of flavonoe in *Zanthoxylum bungeanum* leaves was determined using the aluminum nitrate color developing method. A total of 1 g of *Zanthoxylum bungeanum* leaves powder was weighed and placed into a 100 mL beaker, 50 mL 30% ethanol was added, extracted for 30 min via an ultrasonically assisted extraction method, filtered under reduced pressure, and transferred into a 50 mL volumetric bottle with 30% ethanol. A pipette gun was used to absorb 1 mL sample extract into a 10 mL test tube, and 0.3 mL 5% sodium nitrite solution, 0.3 mL 10% aluminum nitrate solution, and 4 mL 4% NaOH solution were added, respectively. After adding each reagent, the mixture was shaken well and left to sit at room temperature for 6 min, and finally distilled water was added up to 10 mL. After shaking, the absorbance was measured at 510 nm at room temperature for 12 min, and no rutin solution was used as a blank control. Rutin solution with different concentrations (0~0.07 mg/mL) was configured to make a standard curve. The standard curve was drawn with rutin solution, with different concentrations as the horizontal coordinate and absorbance as the vertical coordinate, and the regression equation was established as $y=11.219x-0.0017$, $R^2=0.9991$. The flavonoid content in *Zanthoxylum bungeanum* leaves was calculated using the standard curve.

8. *Determination of crude polysaccharide content*

In this experiment, the content of crude polysaccharide in *Zanthoxylum bungeanum* leaves was determined as follows. A total of 1.0g *Zanthoxylum bungeanum* leaves powder was weighed in a 100 mL beaker, and 50 mL distilled water was added to soak it overnight. Then, the filtrate was placed in an 80°C water bath for 4 h, then the filtrate was extracted again with 50 mL distilled water, and then the two filtrates were combined into a 100 mL volumetric bottle and the volume was set. After shaking well, 1 mL of extract solution was absorbed into a 15 mL test tube and the volume was set to the scale line. The drawing of the standard curve was determined via the anthrone–sulfuric acid method. A total of 0.01g galactose standard was accurately weighed and fully dissolved in a beaker with distilled water, and the volume was fixed to 100 mL to prepare a 0.1mg/mL galactose standard solution. Different volumes of galactose standard liquid were accurately removed into a test tube with a plug and diluted to 2.0 mL with distilled water. A total of 5.0 mL 0.2% anthrone–sulfuric acid solution was added to the mixture and then bathed in water at 70°C for 20 min. The absorbance was measured at 484 nm after cooling to a normal temperature. With the concentration of galactose of different concentrations (which had a good linear relationship in the range of 0-0.06 mg/mL) as the horizontal coordinate and the absorbance value as the vertical coordinate, the regression equation was obtained as y=2.7475x-0.0035 and the correlation coefficient as R^2=0.9971. The absorbance of polysaccharide aqueous solution of *Zanthoxylum bungeanum* leaves was introduced into the regression equation to calculate the crude polysaccharide content of *Zanthoxylum bungeanum* leaves.
